# Novel field observations of coral reef fishes feeding on epiphytic and epizoic organisms associated with the allelopathic seaweed *Galaxaura divaricata*

**DOI:** 10.1002/ece3.9529

**Published:** 2022-11-27

**Authors:** Carolin Nieder, Chen‐Pan Liao, Chen‐Lu Lee, Kendall D. Clements, Shao‐Lun Liu

**Affiliations:** ^1^ Institute of Marine Science University of Auckland Auckland New Zealand; ^2^ Department of Biology National Museum of Natural Science Taichung Taiwan; ^3^ Institute of Marine Biology National Taiwan University Keelung Taiwan; ^4^ School of Biological Sciences University of Auckland Auckland New Zealand; ^5^ Department of Life Science & Center for Ecology and Environment Tunghai University Taichung Taiwan

**Keywords:** coral reef algae, coral reef habitat degradation, epiphytes, fish‐feeding behavior, food source, resilience

## Abstract

In degraded coral reef ecosystems, allelopathic macroalgae have received increasing attention from marine ecologists because their secondary metabolites (also known as allelochemicals) kill corals that grow adjacent to them and weaken the recovery of degraded reefs. One well‐known coral‐killing macroalga is the calcareous red seaweed *Galaxaura*. However, our knowledge of how coral reef fishes interact with allelopathic algae like *Galaxaura* is very limited. Here, we documented novel observations of feeding interactions of 17 species of coral reef fishes (herbivorous and carnivorous) with the filamentous *Galaxaura divaricata* on degraded lagoon patch reefs in Dongsha Atoll (South China Sea). Video analyses showed that territorial farming damselfishes (i.e., *Dischistodus perspicillatus*, *D. prosopotaenia*, *Hemiglyphidodon plagiometopon*, *Pomacentrus grammorhynchus*, *P. adelus*, and *Neoglyphidodon nigroris*) and juvenile parrotfishes (*Scarus schlegeli*, *S. ghobban*, *S. rivulatus*, and *Chlorurus spilurus*) likely used *G. divaricata* as a feeding substratum. Further, microscopic analyses revealed that the filamentous surface of *G. divaricata* harbored a wealth of epiphytic microalgae, such as filamentous cyanobacteria (i.e., *Leptolyngbya*, *Lyngbya*, *Rivularia*, *Oscillatoria*, and *Stigonema*), diatoms (i.e., *Synedra*, *Nitzschia*, *Mastogloia*, and *Pleurosigma*), and filamentous red algae (i.e., *Heterosiphonia*), suggesting that these fishes targeted the nutrient‐rich microscopic epiphytes rather than the nutrient‐poor host. Juvenile benthic carnivores (i.e., Labridae, *Parupeneus multifasciatus*, and *Meiacanthus grammistes*) form feeding assemblages with roving parrotfishes to feed on small invertebrates (i.e., amphipods, copepods, isopods, gastropods, and polychaetes) associated with *G. divaricata*. Given that coral reef fishes appear to target the epiphytes associated with *Galaxaura* rather than the alga itself, these observations thus substantiate the threat posed by the overgrowth of *G. divaricata* to coral recovery in degraded reef systems due to the lack of natural grazers.

## Graphical abstract

Chemical‐rich allelopathic macroalgae are competing with corals in sub‐/tropical reef ecosystems. According to the prevailing view, these algae deter herbivores with their secondary metabolites. Contrary to this view we observed that allelopathic macroalgae like *Galaxaura divaricata*  can provide a substratum for nutrient‐rich epiphytes that in turn present a food source for coral reef fishes. These observations suggest that *Galaxaura* may play an ecological role that has been overlooked and deserves our attention in the context of the resilience of coral reef ecosystems.

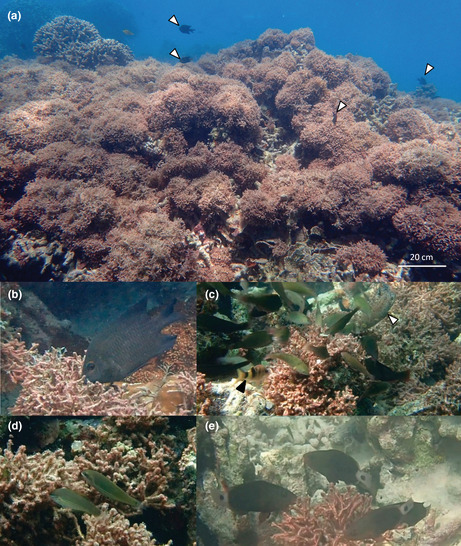

## 1 INTRODUCTION

Coral reefs have changed rapidly over the past decades, often resulting in degraded habitats dominated by macroalgae (Bellwood et al., 2004; Done, 1992; Hughes et al., 2007). More studies are needed to reveal how coral reef fish respond to such habitat degradation (Chong‐Seng et al., 2014; McCormick et al., 2017). In degraded reef ecosystems, allelopathic macroalgae have received increasing attention from marine ecologists because their secondary metabolites (also known as allelochemicals) kill corals that grow adjacent to them and weaken the recovery of degraded reefs (Dixson & Hay, 2012; Rasher et al., 2011). One well‐known coral‐killing macroalga is the red calcareous seaweed *Galaxaura* (Rasher & Hay, 2010a, 2010b). *Galaxaura* thrives in shallow marine waters of the warm temperate, subtropical, and tropical regions of the Atlantic, Pacific, and Indian Oceans (Huisman, Harper, & Saunders, 2004; Huisman, Sherwood, & Abbott, 2004; Liu et al., 2013) and is ubiquitous on coral reefs (McCormick et al., 2017). *Galaxaura* produces allelochemicals that suppress the growth and photosynthetic activity of corals (Rasher et al., 2010a) and prevent the settlement of new coral larvae, hampering postdisturbance coral recovery (Bonaldo & Hay, 2014). Moreover, secondary metabolites in *Galaxaura* are found to deter some corallivorous (Brooker et al., 2017) and herbivorous fishes (Loffler et al., 2015a, 2015b; Rasher & Hay, 2014) from feeding on corals and macroalgae that grow in proximity.

In a recent coral reef survey in the lagoon of the remote Dongsha Atoll (Taiwan), we documented an overgrowth of the filamentous species *Galaxaura divaricata* on several lagoon patch reefs, covering 16%–41% of the substratum (Nieder et al., 2019). Dongsha is the only large coral reef atoll (>500 km^2^) in the northern South China Sea and represents a hotspot for marine biodiversity in this region (Huang et al., 2015). *G. divaricata* is poisonous to corals (Nieder et al., 2019) and its calcareous thallus offers low nutritional value for herbivores. On these shallow (2–12 m depth) lagoon patch reefs, the percent cover of *G. divaricata* remained high year‐round and prevailed for periods of 16 months to over 3.5 years (Nieder et al., 2019). Previous field studies and feeding experiments showed that *Galaxaura* is sparingly consumed by macroalgae‐eating herbivores such as Siganidae (rabbitfishes), Acanthuridae (surgeon fishes), and Kyphosidae (sea chubs) likely due to its poor nutritional value and production of secondary metabolites (Mantyka & Bellwood, 2007a, 2007b; Rasher & Hay, 2014; Rasher et al., 2013). However, many such feeding selectivity experiments use transplanted macroalgae, i.e., presenting different types of seaweeds in a “cafeteria style” array in habitats where these seaweeds may be uncommon (Mantyka & Bellwood, 2007a, 2007b; Rasher et al., 2013). Fish are generally opportunistic and will often try novel foods when these suddenly appear in their habitat (Wulff, 2021). The conclusion that such foods are part of their natural diet is therefore problematic. Feeding experiments in cages prevent fish from foraging normally (Burkepile & Hay, 2008), and under such unnatural circumstances, fish can switch to consume less‐preferred foods. Many seaweed choice studies remove the epiphytes from the macroalgae under investigation prior to experiments (e.g., Kirsch et al., 2002; Lee et al., 2015), and so do not test diet selectivity as it pertains in the wild. During our ecological surveys on *G. divaricata* in the lagoon of Dongsha Atoll, we unexpectedly observed several herbivorous fishes and benthic carnivores in close contact and interacting with *Galaxaura*. Clearly, our observations indicate that there is a lack of knowledge on how coral reef fishes interact with *G. divaricata* in these degraded reefs under natural conditions.

The aims of this study are to (1) document novel in‐situ observations of coral reef fishes feeding on *G. divaricata* on a degraded coral reef using video recordings, and (2) provide examples that these fishes frequently interact with *G. divaricata* likely by targeting the associated microscopic epiphytes and small invertebrates. We hope that the behavioral observations reported here may stimulate future studies to verify that these fishes ingest the epiphytes and small fauna associated with *Galaxaura*.

## 2 METHODS

### 2.1 Study sites

**FIGURE 1:**
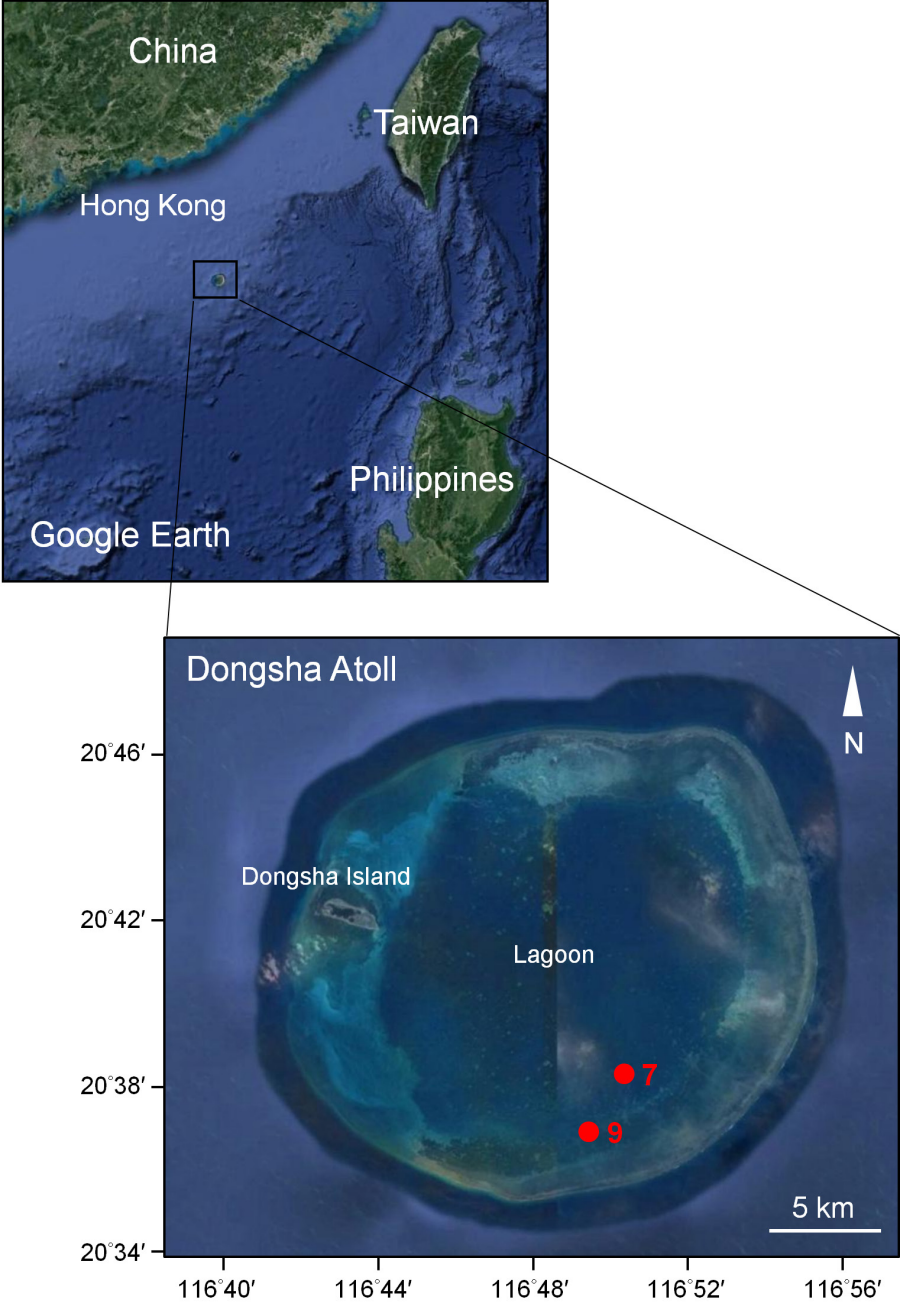
Site map showing the study locations (reef slope no. 7 and reef flat no. 9) on two degraded coral patch reefs inside the lagoon of Dongsha Atoll, South China Sea.

We documented feeding interactions of coral reef fish with the filamentous calcareous red macroalga *Galaxaura divaricata* at two locations of degraded coral reef in the lagoon of Dongsha Atoll, South China Sea in September 2017 (Figure 1). The two study locations, namely the slope of patch reef no.7 (lat. 20°38′24″, long. 116°50′20.999″) and the flat of patch reef no. 9 (lat. 20°36′52.86″, long. 116°49′24.179″) were chosen based on benthic surveys conducted previously on the shallow flats (depth 2–3 m) and the deeper slopes (depth 5–8 m) of twelve coral patch reefs across the Dongsha lagoon (Nieder et al., 2019). According to these surveys the slope of patch reef no.7 and the flat of patch reef no. 9 had low scleractinian coral cover (<10%) and high macroalgal cover (>25%) (Nieder et al., 2019). In addition, *Galaxaura divaricata* was dominant year‐round, covering 16%–41% of the substratum for periods of at least 16 months to over 3.5 years at both locations (Nieder et al., 2019).

### 2.2 Video recordings of feeding behavior

To quantify interactions of reef fishes with *Galaxaura,* we deployed a total of five BenQ SP2 underwater cameras on the reef slope (no. 7) and three on the reef flat (no. 9). All recordings were done between 10 am and noon over a period of 3 days. After deployment, the cameras were left alone and recollected. Although we aimed to film the fish behavior for about 40 min at each camera station, on a few occasions cameras shut off earlier or were displaced by waves, resulting in a shorter recording time than expected. Overall, the recording length ranged between 12.4 and 41.3 min (29.4 ± 9.8 min). The first and last 3 min were omitted from the analysis to allow settling time after camera deployment and to avoid disturbance caused by the collection of the equipment. After camera deployment, we swam away for other ecological surveys. Thus, no divers were present during the recordings. We analyzed 91.2 min of video footage for the reef flat (location no. 9, *N* = 3) and 143.8 min for the reef slope (location no. 7, *N* = 5). Only fishes that were present within the boundaries of a designated area (50 × 50 cm^2^) located 30 cm in front of each camera were included in the analysis. When a fish targeted (or interacted with) naturally growing *G. divaricata* in the video, we identified the species and noted the approximate developmental stage (juvenile and adult) based on visual judgments of the size and coloration of the fish. The following behaviors were counted: bites (by herbivores), strikes (by carnivores), chases after intruding fish, and inspections. A bite was counted when an herbivorous fish directly contacted *Galaxaura* with its mouth, followed by chewing movements, without removing any visible parts of the alga. We would expect to have detected any algal removals of more than 0.5 cm in size, as we were able to detect bite marks in previous feeding experiments with other algae where we used a similar distance between the camera and the feeding fish (unpublished data). A strike was counted when a predatory fish pecked at *Galaxaura* without removing any visible parts of the alga. A chase was counted when a fish defended its territory by chasing an intruding fish from a *Galaxaura* patch. An inspection occurred when a fish hovered a few mm in front of *Galaxaura* for a minimum of 3 s, without striking or biting at *Galaxaura*. The fish were untagged and swam freely in and out of the view of the camera. Therefore, it was not possible to distinguish between individuals. Because we only counted bites/strikes that happened inside our observational areas and not every species was present for the same amount of time, these rates do not represent true estimates for bite/strike rates for the different fish species. Using this method, we were able to capture the behavior of territorial fish species, i.e., farming damselfish, whose territory happened to overlap with our experimental areas.

### 2.3 Observations of juvenile parrotfishes

We were unable to obtain video footage of roving parrotfishes using the stationary camera deployment method described above and therefore chose to follow the parrotfish groups to document their feeding interactions with *Galaxaura*. Because fish may respond to be followed by divers, we first spend at least 10 min on the sites without following the parrotfish groups and observed their feeding behavior from afar. The groups moved continuously, between different patches of *Galaxaura*, spending between 1 and 5 s feeding on each patch. The parrotfishes did not appear to be affected by the presence of the divers. The two divers followed each different group of parrotfishes for a three‐minute acclimation period and then recorded the group for 1–2 min by keeping a two‐meter distance. Each diver repeated this process at least five times at each site over the course of 3 days. No considerable difference in behavior by the fish was noticed during recordings. Like before, the fish spent 1–5 s feeding on a *Galaxaura* patch before moving to the next. We therefore concluded that the parrotfish‐feeding behavior filmed here can be viewed as natural and that any effect of the divers is negligible. Multiple groups of roving juvenile parrotfishes were on site on each observational day; however, because these fish were not tagged, we cannot exclude the possibility that one group may have been followed more than once. It was not possible to quantify bite rates from the parrotfish recordings because it was difficult to track individuals, as parrotfishes moved too quickly within their group and between *Galaxaura* patches.

### 2.4 Microscopic epiphytes associated with *Galaxaura*

To examine microscopic epiphytes associated with *G. divaricata*, we haphazardly collected 15 *Galaxaura* specimens from the same depths and habitats where the video observations were made and preserved in 5%–10% formalin‐seawater. The types of microscopic epiphytes are highly variable among different sections (or positions) of the thallus. It is therefore difficult for us to undertake quantitative assessments. Instead, we observed microscopic epiphytes on the branches where the fish interacted and provide examples of common microepiphytes associated with *G. divaricata* to which the fish might target. The specimens were decalcified in 1% HCl solution, and either squashed or sectioned by hand. Photomicrographs were taken on a Pixera Penguin 600CL digital camera and a Nikon 995 digital camera. Examined specimens were deposited at the herbarium of the Department of Life Science, Tunghai University (THU), Taiwan.

### 2.5 Invertebrate fauna associated with *Galaxaura*

To quantify the invertebrates associated with *G. divaricata*, we haphazardly collected 30 thalli (diameter range 5–25 cm) and counted the invertebrates using the following categories: crabs, foraminifera, gastropods, gastropod egg capsules, isopods, mollusks, polychaetes, shrimps, sponges, and tunicates. We measured the total length or greatest diameter of each organism; and, those measuring <1 mm were excluded from the analysis.

### 2.6 Statistical analyses

Data from our stationary video recordings were analyzed using multivariate Poisson mixed‐effect models. The number of events for the *Galaxaura* bite/strike, the intruder chase, and the *Galaxaura* inspection was fitted against “reef area” (flat and slope), “fish type” (herbivore and predator), and their interaction. We included a random intercept for each fish species and a random slope for reef area and fish species in the models. To compare the number of events per time interval among different videos, the video length (min) was naturally log‐transformed and treated as an offset term in the Poisson models. Specifically, the offset term is to normalize the recording time length. Priors were assigned for fixed effects (2×‐scaled student‐t distribution, 3 degrees of freedom) and for standard deviations of random effects (5×‐scaled half student‐t distribution, 5 degrees of freedom). Models were fitted with a Markov Chain Monte Carlo (MCMC) based algorithm using the R package “brms” (ver.2.12.0; Bürkner, 2017; Table S1). Each MCMC chain included 10k burn‐in and 10k posterior iterations. Highest density intervals (HDI) of a log‐rate ratio posterior between two groups were evaluated according to the Bayesian model. The parameters estimated the following variables: intercept (mean counts per minute); reef area (flat vs. slope); fish type (herbivore vs. predator); interaction (average among herbivore in reef flat and predator in reef slope vs. average among predator in reef flat and herbivore in reef slope). We tested the significance of the probability of direction (Pd) and Bayes factor (BF) by using the Savage–Dickey density ratio method (Verdinelli & Wasserman, 1995). We found no significant interactions between reef area and fish type (Bayes factor < 1). Pairwise multiple comparisons among groups were done by using equivalence tests, where a significant difference was only assumed when a HDI fell outside the region of practical equivalence (ROPE) (Kruschke & Liddell, 2018).

## 3 RESULTS

**FIGURE 2:**
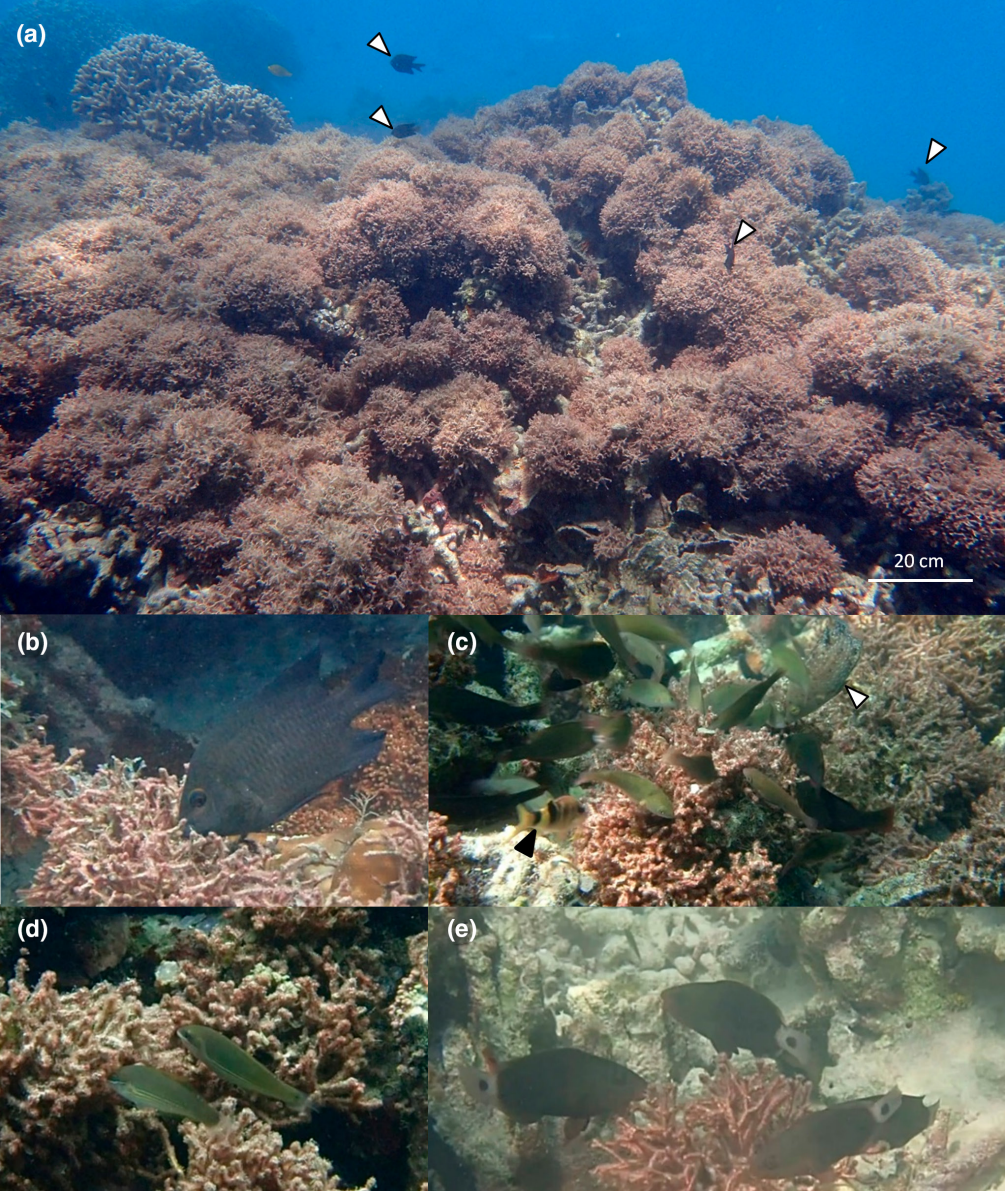
Photographs showing the overgrowth of *Galaxaura divaricata* on a degraded coral reef flat in the lagoon of Dongsha Atoll with three types of coral reef fishes (territorial farming damselfish, juvenile parrotfish, and carnivorous fish that use *Galaxaura divaricata* as feeding substratum. (a) Territorial farming damselfishes (white arrowheads) defend their territories against intruders across the *Galaxaura* canopy; (b) farming damselfish *Hemiglyphidodon plagiometopon*, likely feeding on epiphytes and detritus from the surface of *G. divaricata*; (c) a group of juvenile parrotfishes including *Scarus rivulatus*, *S. ghobban*, *S. schlegelii*, and *Chlorurus spilurus* likely feeding on epiphytic microalgae from the surface of *G. divaricata*. Predators including goatfish, *Parupeneus multifasciatus* (black arrowhead), and the wrasse *Cheilinus chlorourus* (white arrowhead) follow a group of roving parrotfish to feed on small invertebrates associated with *G. divaricata*; (d) the carnivorous wrasse, *Stethojulis strigiventer* forage for small invertebrates hidden between the branches of *G. divaricata*; (e) late juvenile or early initial phase *Chlorurus spilurus* likely grazing on microepiphytes from the surface of *G. divaricata*.

**FIGURE 3:**
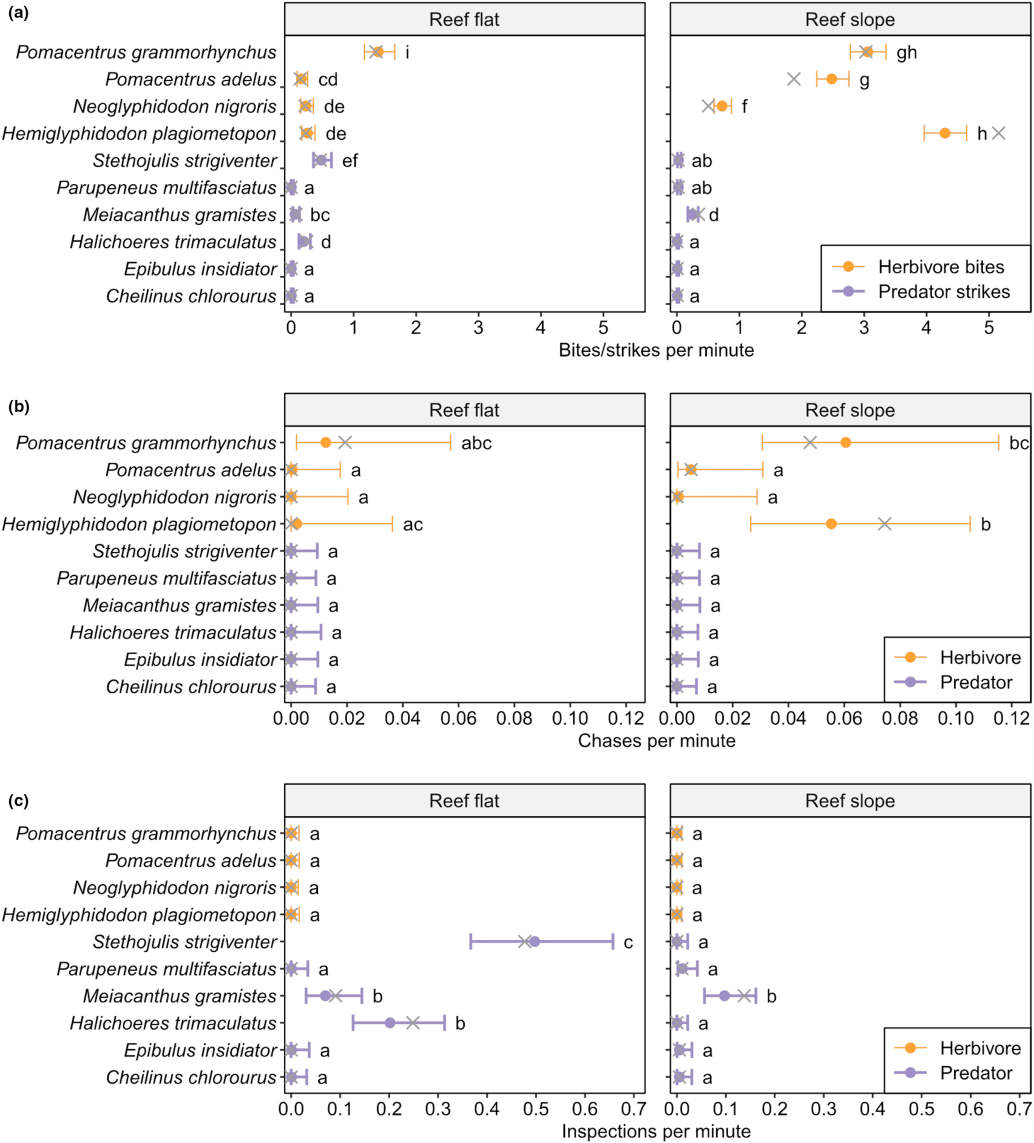
Rates at which *Galaxaura divaricata* was targeted by different reef fishes. (a) Herbivore bite rate (counted when a herbivorous fish directly contacted *Galaxaura* with its mouth, followed by a chewing motion, without removing any visible parts of the alga) and predator strike rate (counted when a carnivorous fish pecked at *Galaxaura*, without removing any visible parts of the alga); (b) chase rate against intruders (counted when a fish attacked an intruding fish and chased it away from a *Galaxaura* patch); (c) inspection rate (defined as close‐up search by a fish a few mm in front of *Galaxaura* and for more than 3 s, without the occurrence of any bites or strikes). Filled dots indicate the median; X indicates the mean; whiskers mark 95% density intervals. The letters next to the whiskers show the results of the pairwise multiple comparisons. Groups without a common letter are considered significantly different.

We identified a total of 17 species of coral reef fishes of various food preference types (microphage, herbivore, omnivore, detritivore, and carnivore) that were likely feeding from the surface of *G. divaricata* (Table 1 and Figure 2). Firstly, using the stationary video recordings we identified four species of territorial farming damselfish, including *Hemiglyphidodon plagiometopon* (Figure 2b), *Pomacentrus grammorhynchus*, *P. adelus*, and *Neoglyphidodon nigroris* that interacted with *G. divaricata* (Figure 3). Of the four species, *H. plagiometopon* and *P. grammorhynchus* were observed to target *G. divaricata* most frequently with an average of 3–5.2 bites per minute (Figure 3a; and Table S1). During feeding the damselfishes contacted *G. divaricata* without removing any visible parts of the alga itself (Videos S1, S2, and S3), suggesting that these fish likely target small epiphytes, detritus, and animal materials on the surface of *G. divaricata*. The damselfish defended the observed *G. divaricata* patches against intruding fish that fed on *G. divaricata* inside their territory (Figure 3b; Videos S4‐S7). We also identified six carnivorous species that inspected and struck at *G. divaricata* (Figure 3c), including juvenile Labridae, *Cheilinus chlorourus*, *Halichoeres trimaculatus*, *Stethojulis strigiventer* (Figure 2d), *Epibulus insidiator*, blenny *Meiacanthus grammistes*, and juvenile goatfish *Parupeneus* multifasciatus. Overall, the bite/strike, chase, and inspection rates were mainly determined by the fish type (herbivore vs. predator) (Pd = 94%–99%; BF = 1.76–6.16) rather than reef type (flat vs. slope) (Pd = 57%–83%; BF = 0.06–0.93) or interaction between fish type and reef type (Pd = 54%–83%; BF = 0.12–0.97). Herbivorous bite rate was about 29 times higher than the predatory strike rate (rate ratio, 29.3; Table S1a). Chase rate (i.e., chase of intruding fish rate) for herbivores was about 24 times higher than the chase rate for predatory fish (rate ratio, 24.3; Table S1b). Herbivores rarely inspected *G. divaricata* (without subsequent biting), which was frequently observed in carnivorous species (rate ratio, 0.02; Table S1c).

**TABLE 1:** Species of coral reef fishes that were observed to feed on the epiphyte and invertebrate community associated with *Galaxaura divaricata* on degraded patch reefs in the lagoon of Dongsha Atoll (South China Sea).

Family	Species	Stage	Feeding type	Dietary targets	Reference
Scaridae	*Chlorurus spilurus* (Valenciennes, 1840)	Juvenile	Microphage	Cyanobacteria and microalgae	Clements et al. (2017)
Scaridae	*Scarus ghobban* (Forsskål, 1775)	Juvenile			Clements et al. (2017)
Scaridae	*Scarus rivulatus* (Valenciennes, 1840)	Juvenile			Nicholson and Clements (2020)
Scaridae	*Scarus schlegeli* (Bleeker, 1861)	Juvenile			Clements et al. (2017)
Pomacentridae	*Dischistodus perspicillatus* (Cuvier, 1830)	Adult	Algivore, omnivore, and farmer	Macroalgae and microalgae (i.e., diatoms, cyanobacteria, and detritus)	Wilson and Bellwood (1997)
Pomacentridae	*Dischistodus prosopotaenia* (Bleeker, 1852)	Adult			Wilson and Bellwood (1997)
Pomacentridae	*Hemiglyphidodon plagiometopon* (Bleeker, 1852)	Juvenile and adult			Wilkinson and Sammarco (1983)
Pomacentridae	*Pomacentrus adelus* (Allen, 1991)	Juvenile and adult			Ceccarelli (2007)
Pomacentridae	*Pomacentrus grammorhynchus* (Fowler, 1918)	Adult			Emery and Thresher (1980)
Pomacentridae	*Neoglyphidodon nigroris* (Cuvier, 1830)	Juvenile and adult			Ceccarelli (2007)
Pomacentridae	*Stegastes fasciolatus* (Ogilby, 1889)	Juvenile			Cardona and Clayton (1999); Feitosa et al. (2012)
Labridae	*Epibulus insidiator* (Pallas, 1770)	Juvenile	Benthic carnivore	Crustaceans	Sano (1984)
Labridae	*Cheilinus chlorourus* (Bloch, 1791)	Juvenile		Mollusks and crustacean	Sano (1984)
Labridae	*Halichoeres trimaculatus* (Quoy, Gaimard, 1834)	Juvenile		Crustaceans (i.e., copepods)	Kramer et al. (2016)
Labridae	*Stethojulis strigiventer* (Bennett, 1833)	Juvenile		Crustaceans (i.e., copepods)	Kramer et al. (2016)
Blenniidae	*Meiacanthus grammistes* (Valenciennes, 1836)	Juvenile and adult		Zooplankton, small invertebrates	Hundt et al. (2014)
Mullidae	*Parupeneus multifasciatus* (Quoy, Gaimard, 1825)	Juvenile		Crustaceans, crabs, shrimp, polychaetes, bivalve mollusks, amphipods, and gastropods	Myers (1999)

Second, using the video footage of the schools of roving juvenile parrotfishes, we identified four species that targeted *G. divaricata*, including *Chlorurus spilurus*, *Scarus ghobban*, *S. rivulatus*, and *S. schlegeli* (Figure 2c,e; Videos S3–S5, and S7). The schools numbered 10–25 individuals and fed for approximately 3–5 s on a patch of *G. divaricata* before moving to another. Like the damselfish, the juvenile parrotfishes did not seem to ingest *G. divaricata* itself, rather they appeared to scrape material from the surface. On several occasions we observed juvenile *Parupeneus multifasciatus* and *Cheilinus chlorourus* following schools of foraging parrotfishes, likely preying on small animals disturbed by the roving parrotfishes (Figure 2c; Videos S4 and S7).

**FIGURE 4:**
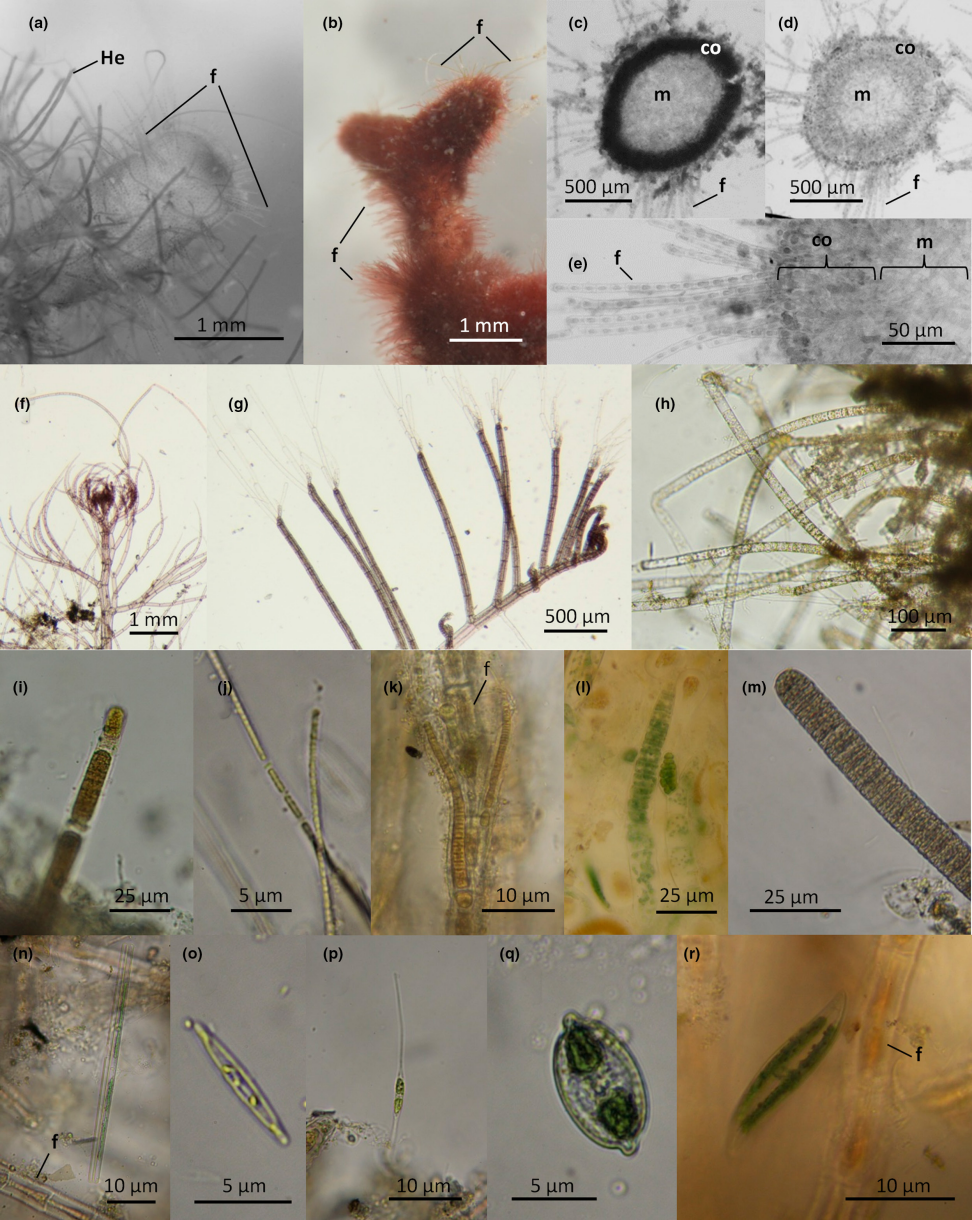
Examples of epiphytic microalgae that accumulate among the assimilatory filaments of *Galaxaura divaricata*, potentially providing nutrient‐rich food for herbivorous fishes. (a) Sun‐exposed, and (b) shaded terminal branches of *G. divaricata* with assimilatory filaments and epiphytic filamentous red alga, *Heterosiphonia* sp. cross‐section of a *G. divaricata* branch showing the inner medulla and the outer cortex (c) before, and (d) after decalcification. (e) Close‐up view of assimilatory filaments, cortical layer, and medulla of *G. divaricata*. Different groups of microepiphytes that grow on *G. divaricata* filaments are shown as follows: Filamentous red algae, (f) *Heterosiphonia* sp1., and (g) *Heterosiphonia* sp2.; (h) filamentous green alga, *Ulothrix* sp.; filamentous cyanobacteria: (i) *Lyngbya* sp1., (j) *Leptolyngbya* sp2, (k) *Rivularia* sp., (l) *Stigonema* sp., and (m) *Oscillatoria* sp.; diatoms: (n) *Synedra* sp., (o) *Synedra* sp., (p) *Nitzschia closterium*, (q) *Mastogloia* sp., and (r) *Pleurosigma* sp. abbreviations: (f) G*. divaricata* assimilatory filaments; (co) outer cortex; (He) *Heterosiphonia*; (m) inner medulla.

The thallus of *G. divaricata* has numerous bifurcating branches made of an inner less‐calcified medulla, and an outer strongly calcified cortical cell layer (Figure 4c,d). The calcareous branches are covered with dense microscopic filaments (~4 μm wide, ~500 μm long, 100 filaments per 1 mm^2^; see example in Figure 4a,b,e). Observations under the microscope revealed that the surface of *G. divaricata* is heavily colonized by a variety of microscopic epiphytes (Figure 4f–r), i.e., filamentous red algae (i.e., Gelidiales and Ceramiales, *Heterosiphonia* sp.), unicellular and filamentous green algae (*Ulothrix* sp.), cyanobacteria (*Leptolyngbya* sp., *Lyngbya* sp., *Rivularia* sp., *Oscillatoria* sp., *Stigonema* sp.) and diatoms (*Synedra* sp., *Nitzschia closterium*, *Mastogloia* sp., and *Pleurosigma* sp.). In terms of small invertebrates (<1 cm in length), we found that foraminifera, amphipods, copepods, crabs and other crustaceans, and gastropods were most frequently associated with *G. divaricata* (Table 2; Figure S1). Gastropod egg capsules and tunicates were often directly attached to the branches of *G. divaricata*.

**TABLE 2:** Count, occurrence frequency, and size measurements of small epizoic invertebrates associated with *Galaxaura divaricata* (*n* = 30) on degraded coral patch reefs in the lagoon of Dongsha Atoll (South China Sea).

Invertebrate	Count	Occurrence frequency (%)	Size^a^ (mm)
Foraminifera	1009	100	2 ± 1
Tunicate	30	100	8 ± 2
Gastropod egg capsule	25	83	5 ± 3
Amphipods and copepods	20	67	4 ± 2
Other crabs	15	50	5 ± 3
Gastropod	13	43	5 ± 4
Sponge	12	40	6 ± 5
Brittle star	6	20	3 ± 2
Other crustaceans	5	16	8 ± 2
Hermit crab	4	13	3 ± 1

^a^ Size stands for the total length or the longest diameter of the target organisms.

## 4 DISCUSSION

Understanding how different groups of coral reef fish interact with dominant macroalgae on degraded reefs provides valuable insight into resource use in these systems (Chong‐Seng et al., 2014). The allelopathic calcareous macroalgae of the genus *Galaxaura* are common on coral reefs, yet we do not understand how coral reef fish interact with these algae. According to the prevailing view, *Galaxaura* deters herbivores with its arsenal of secondary metabolites (Bonaldo & Hay, 2014; Loffler et al., 2015a, 2015b; Rasher & Hay, 2010a, 2010b). Contrary to this view we observed feeding interactions of 17 species of coral reef fish, including juvenile parrotfish, territorial farming damselfish, and juvenile benthic carnivores, with the filamentous *Galaxaura divaricata* on degraded patch reefs in the lagoon of Dongsha Atoll, South China Sea.

It is often assumed that when fish feed on a macroalga that it is the alga they are eating. This view led to the perception that parrotfishes eat macroalgae, including turf (e.g., Adam et al., 2015; Feitosa & Ferreira, 2015; Pereira et al., 2016). Our feeding observations indicate that while juvenile parrotfishes, i.e., *Scarus schlegeli*, *S. ghobban*, *S. rivulatus*, and *Chlorurus spilurus*, took many small bites from *G. divaricata*, they did not appear to consume the thallus itself. Instead, they likely primarily targeted epiphytic cyanobacteria and microscopic algae (Clements et al., 2017; Nicholson & Clements, 2020) on *G. divaricata*, consistent with studies on juvenile parrotfish in other systems (Bellwood, 1988; Chen, 2002; Feitosa & Ferreira, 2015). Similar feeding behavior has also been reported in adults of some of these species, where they fed on epiphytes growing on the brown algae *Sargassum* rather than on the *Sargassum* itself (Lefevre & Bellwood, 2010; Lefèvre & Bellwood, 2011; Verges et al., 2012). Parrotfishes target foods that are rich in protein and lipid, especially microscopic photoautotrophs (cyanobacteria and microscopic algae) that colonize other macroalgae, turf algae, crustose coralline algae, and dead coral substratum (Clements et al., 2017; Clements & Choat, 2018; Nicholson & Clements, 2020). The juvenile parrotfish in the present study may incidentally ingest small amounts of *G. divaricata* during feeding; however, they likely obtain limited nutritional benefit from it.

All damselfish species that used *G. divaricata* as a feeding substratum in this study (Table 1) are territorial farmers. Compared with bite rates of damselfish for other algae reported by Ceccarelli (2007), our data are much lower. Given that we have normalized the recording time by treating it as an offset term in our Poisson models (see section 2 for details), one possible explanation for the difference between previous observations and our observations is likely that the natural feeding territories of the damselfishes are much larger than the small observational areas used in our stationary video recordings. As a result, the bite rates reported here may underestimate the feeding rates for these species. Farming damselfishes influence the algal community in their territory by cultivating selected algae (Brawley & Adey, 1977; Ceccarelli, 2007; Wilkinson & Sammarco, 1983). Our observations suggest that these damselfish use *G. divaricata* as a source of epiphytic macroalgae (i.e., *Acanthophora spicifera*, *Ceramium*, *Coelothrix*, Gelidiales, *Hypnea*; Nieder et al., 2019) and microalgae (i.e., diatoms and cyanobacteria) which they consume and aggressively defend against intruders. None of the damselfish appeared to ingest *G. divaricata* itself. Similar relationships were demonstrated for farming damselfishes that eat epiphytes from the surface of *Sargassum* but not *Sargassum* itself (Ceccarelli et al., 2005). Because farming damselfishes mediate algal diversity (Hixon & Brostoff, 1983) and structure within their territories (Ceccarelli, 2007; Ceccarelli et al., 2005), these fish species are likely to influence the epiphyte community on *G. divaricata* through their feeding and farming behavior.

It was noticeable that the juvenile parrotfishes preferred grazing on *G. divaricata* mats than on turf, dead coral, and rubble, suggesting that the microepiphyte community growing on *G. divaricata* is distinct and of greater nutritional value. The calcareous and filamentous branches of *G. divaricata* provide a complex substratum for a variety of epiphytic macroalgae (Nieder et al., 2019) and microalgae (this study), i.e., cyanobacteria, diatoms, unicellular green algae, and small filamentous red algae (Figure 3). Heavy colonization by cyanobacteria and other microalgae has been reported in other coral reef macroalgae including *Dictyota*, *Lobophora*, *Padina*, *Halimeda*, and *Sargassum* (Fricke et al., 2011; Hensley et al., 2013; Penhale & Capone, 1981; Stanca & Parsons, 2021).

A recent study showed that environmental factors (i.e., temperature and wave height), location, seasonality, and type of hosts all were important factors in determining the epiphytic microalgal community on tropical seaweeds (Stanca & Parsons, 2021). The dense hair‐like filaments of *G. divaricata* may facilitate the accumulation of detritus (particulate organic matter and inorganic matter) and promote the growth of microepiphytes, both of which provide food sources for various groups of herbivores and detritivores.

Small crustaceans including amphipods and copepods were very common on *G. divaricata*. Newly settled parrotfish of under 30 mm in size feed primarily on small crustaceans and undergo a diet shift to mainly photoautotrophs at 15–30 mm TL (Chen, 2002). The juvenile parrotfishes observed in the present study were 10–12 cm and were thus very unlikely to be targeting copepods on *G. divaricata*. However, based on the work by Kramer et al. (2016), it is possible that they ingest copepods incidentally while feeding on primary producers. Diet analysis on these juvenile parrotfishes would be of interest to confirm what materials they are ingesting.

*Galaxaura divaricata* also provides a feeding substratum for various benthic carnivores (Table 1). The juvenile labrids *Halichoeres trimaculatus* and *Stethojulis strigiventer* and the goatfish *Parupeneus multifasciatus* were most frequently observed feeding on *G. divaricata*. These fishes likely target the numerous small invertebrates (i.e., copepods, amphipods, isopods, crabs, gastropods, and polychaetes) that inhabit *G. divaricata. Parupeneus multifasciatus* and *Cheilinus chlorourus* frequently formed foraging associations with the parrotfishes, a feeding strategy well‐known for goatfish and benthic Labridae on coral reefs (Lukoschek & McCormick, 2000; Macarthur & Hyndes, 2007).

*Galaxaura* is infrequently consumed by herbivorous fishes, likely because its high calcium carbonate content that diminishes its nutritional value to most species (Bonaldo & Hay, 2014; Hay, 1986; Littler et al., 2006; Lobel, 1981; Loffler et al., 2015a, 2015b; Mantyka & Bellwood, 2007a). Hence, allelopathic and nutrient‐poor macroalgae such as *G. divaricata* are not targeted by common herbivores (e.g., rabbitfish, surgeon fishes, sea chubs) and once established are likely to dominate reef habitats for several years (Nieder et al., 2019). Previous field studies suggested that herbivorous fishes including parrotfishes are deterred by *Galaxaura* secondary metabolites (Brooker et al., 2017; Mantyka & Bellwood, 2007a, 2007b), and concluded that *Galaxaura* could act as a refuge for more nutrient‐rich macroalgae that grow in close association with it (Loffler et al., 2015a, 2015b). The present study suggests that some farming damselfishes, juvenile parrotfishes, and small benthic carnivores use *G. divaricata* as a feeding substratum. By providing a suitable substratum for primary producers and invertebrates, and by serving as a feeding substratum for various herbivores, detritivores, and small benthic carnivorous fishes, *G. divaricata* serves as a secondary habitat former (Bittick et al., 2018; Thomsen et al., 2016).

The influence of *Galaxaura* secondary metabolites on fishes remains poorly understood (Xu et al., 2008). McCormick et al. (2017) therefore hypothesized that the overgrowth of this alga could change the chemical landscape on degraded reefs, which may impact not only the health of corals but also the sensory perception of reef fishes. For example, corallivores avoided feeding on corals that were in contact with *Galaxaura filamentosa* (Brooker et al., 2017), and laboratory experiments indicated that chemicals from *Galaxaura rugosa* prevent the juvenile Ambon damselfish *Pomacentrus amboinensis* from responding to a predator alarm odor (McCormick et al., 2017). However, our field observations indicate that being in the vicinity of *G. divaricata* may not pose too much of a problem for damselfishes. Further research on the effects of dense *Galaxaura* assemblages, including its epiphytes on communities in degraded coral reefs is needed.

It is important to note that the present study is limited in that it is based on behavioral observations only. The claims made here that the different fish species target the microepiphytes and benthic invertebrates and not necessarily the algae itself need additional support through analyses of stomach/gut contents, dietary DNA metabarcoding, or stable isotopes. Without additional analyses, we cannot rule out the possibility that these fishes may be ingesting very small amounts of *Galaxaura*. For instance, it would be of interest whether these fishes ingest the microscopic hair‐like assimilatory filaments that cover the calcified thallus of *G. divaricata*, and whether these filaments or other parts of *Galaxaura* provide any nutritional benefits for these fishes.

Overall, our observations have two important implications. First, these field observations provide behavioral evidence that different reef fishes likely feed on nutrient‐rich epiphytes and invertebrates associated with *Galaxaura*, rather than removing the nutrient‐poor host algae itself. Thus, this study further supports the view that the coral‐competing *Galaxaura*, if overgrown, can be a serious ecological issue for the prevention of coral recovery in a degraded coral reef ecosystem. Second, small epiphytes and epifauna associated with macroalgae should be considered in feeding selectivity studies, as these may be the primary nutritional target for some fish.

## AUTHOR CONTRIBUTIONS

**Carolin Nieder:** Conceptualization (equal); data curation (equal); formal analysis (equal); investigation (equal); software (equal); visualization (equal); writing – original draft (equal); writing – review and editing (equal). **Chen‐Pan Liao:** Data curation (equal); formal analysis (equal); investigation (equal); methodology (equal); software (equal); validation (equal); visualization (equal); writing – review and editing (equal). **Chen‐Lu Lee:** Data curation (equal); formal analysis (equal); investigation (equal); methodology (equal); software (equal); validation (equal); visualization (equal); writing – review and editing (equal). **Kendall D. Clements:** Conceptualization (equal); writing – review and editing (equal). **Shao‐Lun Liu:** Conceptualization (equal); funding acquisition (equal); investigation (equal); project administration (equal); resources (equal); supervision (equal); validation (equal); visualization (equal); writing – review and editing (equal).

## CONFLICT OF INTEREST

The authors have declared that no competing interests exist.

### OPEN RESEARCH BADGES

This article has earned Open Data, Open Materials and Preregistered Research Design badges. Data, materials and the preregistered design and analysis plan are available at https://github.com/chenpanliao/Coral‐reef‐fish‐feeding‐on‐Galaxaura.

## Supporting information


Appendix S1

## Data Availability

Data available in article Supporting Information.
