# Supplementary material for: Novel field observations of coral reef fishes feeding on epiphytic and epizoic organisms associated with the allelopathic seaweed Galaxaura divaricata
Source: Ecol Evol. 2022 Nov 27;12(11):e9529. doi: 10.1002/ece3.9529 (PMC9702579; doi:10.1002/ece3.9529)
Supplement: Supplementary file 1 — Appendix S1 [file ECE3-12-e9529-s001.zip › GalaxauraFish_SupportingInformation.docx]

**SUPPLEMENTARY MATERIALS**

**Table S1.** Results of fitting (a) bites/strikes per minute, (b) chases per minute and (c) inspections per minute using a multivariate Poisson mixed−effect model. Abbreviations: F, flat; S, slope; H, herbivore; P, predator, HDI, Highest density intervals; Pd, Probability of direction; BF, Bayes factor.

|  | Parameter | Posterior median and 95% HDI | Exponential function of posterior median and 95% HDI | Pd | BF |
| --- | --- | --- | --- | --- | --- |
| (a) | Intercept | −2.21 (−3.65, −1.04) | 0.11 (0.03, 0.35) | — | — |
|  | Reef area (F vs S) | −0.68 (−2.54, 1.14) | 0.51 (0.08, 3.11) | 0.793 | 0.93 |
|  | Fish type (H vs P) | 3.38 (0.83, 5.73) | 29.26 (2.29, 307.96) | 0.992 | 3.08 |
|  | Interaction | −1.36 (−4.30, 1.65) | 0.26 (0.01, 5.23) | 0.828 | 0.97 |
| (b) | Intercept | −7.75 (−11.25, −5.36) | 0.0004 (0, 0.0047) | — | — |
|  | Reef area (F vs S) | −1.19 (−4.15, 1.52) | 0.31 (0.02, 4.55) | 0.829 | 0.06 |
|  | Fish type (H vs P) | 3.19 (−1.14, 8.58) | 24.25 (0.32, 5303) | 0.940 | 1.76 |
|  | Interaction | −1.06 (−5.54, 2.86) | 0.35 (0, 17.41) | 0.721 | 0.12 |
| (c) | Intercept | −7.41 (−10.43, −5.09) | 0.0006 (0, 0.0062) | — | — |
|  | Reef area (F vs S) | 0.24 (−2.98, 3.31) | 1.27 (0.05, 27.29) | 0.565 | 0.68 |
|  | Fish type (H vs P) | −4.11 (−10.11, 0.49) | 0.02 (0, 1.63) | 0.977 | 6.16 |
|  | Interaction | −0.16 (−4.74, 4.22) | 0.85 (0.01, 68.27) | 0.535 | 0.85 |

**
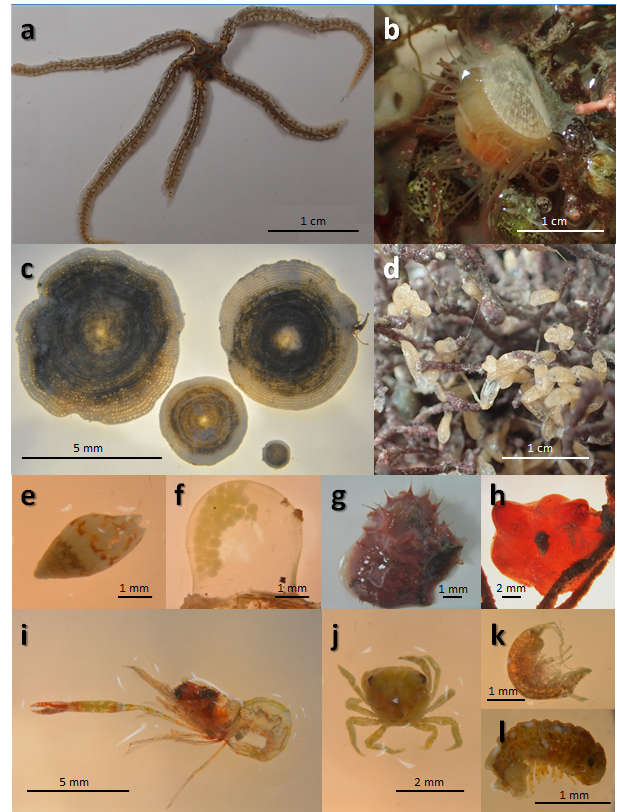
**

**Figure S1.** Examples of epizoic invertebrates associated with *Galaxaura divaricata* potentially providing a food resource for carnivorous reef fishes, including (a) brittle star; (b) bivalve, Veneridae; (c) foraminifera, *Amphisorus hemprichii*; (d and f) gastropod egg capsules; (e) gastropod, Collumbellidae; (g) slug; (h) tunicate; (i) shrimp; (j) crab; (k) amphipod; and (l) isopod.

**Video S1.** The lagoon damselfishes *Hemiglyphidodon plagiometopon* and juvenile *Pomacentrus adelus* feeding on epiphytes on *G. divaricata* on the reef slope (depth 5 m). It is not possible to say what they are eating, but it is likely cyanobacteria, small rhodophytes, detritus, and animal material. Note how *H. plagiometopon* spits out a foraminifer (at 26 sec), which it had picked up from the surface of *G. divaricata* during feeding.

**Video S2.** Juveniles of *Pomacentrus adelus* and *Hemiglyphidodon plagiometopon* feeding from the surface of *G. divaricata* on the reef slope (depth 5 m).

**Video S3.** Late juvenile or early initial phase *Chlorurus spilurus* grazing micro-epiphytes from *G. divaricata* on the reef slope (depth 8 m).

**Video S4.** Schools of foraging juvenile parrotfishes**,** including *Chlorurus spilurus*, *Scarus ghobban*, *Scarus rivulatus*, and *Scarus schlegeli* grazing from the surface of *G. divaricata* on a degraded reef slope (depth 8 m). Amid the parrotfishes is a juvenile goatfish, *Parupeneus multifasciatus* that strikes at *G. divaricata* (at 8 sec) probably to prey on associated invertebrates that were disturbed by the school. Frequent attacks by the territorial damselfish, *Pomacentrus grammorhynchus* can be seen.

**Video S5.** Schools of foraging juvenile parrotfishes**,** including *Chlorurus spilurus*, *Scarus ghobban*, *Scarus rivulatus*, and *Scarus schlegeli* grazing from the surface of *G. divaricata* on a degraded reef slope (depth 8 m). The territorial damselfish, *Dischistodus prosopotaenia* frequently attacks the intruders. Note that these juvenile parrotfishes prefer to graze on *G. divaricata* rather than on bare substrate and rubble.

**Video S6.** *Acropora* rubble area with *G. divaricata* mainly showing *Stethojulis strigiventer* pecking at the *Galaxaura* on a degraded reef flat (depth 2.5 m). These labrids are likely foraging for small crustaceans in *G. divaricata*.

**Video S7.** Showing a school of roving juvenile parrotfishes (i.e., *Chlorurus spilurus*, *Scarus ghobban*, *Scarus rivulatus*, and *Scarus schlegeli*) feeding from the surface of *G. divaricata* on a degraded reef flat (depth 2-3 m). Associated with the parrotfishes can be seen foraging a juvenile goatfish, *Parupeneus multifasciatus* (at 8 sec) and a juvenile wrasse, *Epibulus insidiator* (at 12 sec).

**Video S8.** Showing a school of roving juvenile parrotfishes (i.e., *Chlorurus spilurus*, *Scarus ghobban*, *Scarus rivulatus*, and *Scarus schlegeli*) feeding from the surface of *G. divaricata* on a degraded reef flat (depth 2-3 m). A juvenile goatfish, *Parupeneus multifasciatus* (at 1 sec) and the juvenile wrasse, *Stethojulis strigiventer* (at 20 sec) forage in associating with the parrotfishes. The school is being attacked by the territorial farming damselfish *Neoglyphidodon nigroris*.
